# Psychological Distress, Fear of COVID-19, and Resilient Coping Abilities among Healthcare Workers in a Tertiary First-Line Hospital during the Coronavirus Pandemic

**DOI:** 10.3390/jcm10071465

**Published:** 2021-04-02

**Authors:** Enrico Collantoni, Anna Maria Saieva, Valentina Meregalli, Cristian Girotto, Giovanni Carretta, Deris Gianni Boemo, Greta Bordignon, Alfio Capizzi, Cristina Contessa, Maria Vittoria Nesoti, Daniele Donato, Luca Flesia, Angela Favaro

**Affiliations:** 1 Department of Neurosciences, University of Padua, 35128 Padova, Italy; valentina.meregalli@studenti.unipd.it; 2 Department of Directional Hospital Management, Padua University Hospital, 35128 Padova, Italy; annamaria.saieva@aopd.veneto.it (A.M.S.); cristian.girotto@aopd.veneto.it (C.G.); giovanni.carretta@aopd.veneto.it (G.C.); derisgianni.boemo@aopd.veneto.it (D.G.B.); greta.bordignon@aopd.veneto.it (G.B.); alfio.capizzi@aopd.veneto.it (A.C.); cristina.contessa@aopd.veneto.it (C.C.); mariavittoria.nesoti@aopd.veneto.it (M.V.N.); daniele.donato@aopd.veneto.it (D.D.); 3 Padua Neuroscience Center, University of Padua, 35128 Padova, Italy; 4 Associazione Novilunio Onlus, 35020 Ponte San Nicolò (PD), Italy; luca.flesia@ordinepsicologiveneto.it

**Keywords:** coronavirus, COVID-19, health care workers, mental health, depression, anxiety, insomnia, coping abilities

## Abstract

The COVID-19 pandemic is causing a heavy burden in hospital healthcare workers (HCW) in terms of increased work, organizational changes, risk exposure, and social stigma. The present study aims at evaluating the psychological outcome among HCWs at the final stages of the first wave of the COVID-19 pandemic. This cross-sectional and survey-based study was conducted during June 2020 among 996 HCWs of the University Hospital of Padova. All the subjects completed questionnaires investigating the perception of risk of infecting or being infected by COVID-19, psychopathological variables, and coping abilities. Compared to physicians and healthcare assistants, nurses showed higher levels of depression (*p* = 0.002), insomnia (*p* < 0.001), and generalized anxiety (*p* = 0.001). Females reported increased concerns about the possibility of infecting others (*p* = 0.046), greater anxiety (*p* < 0.001), COVID-19 related fears (*p* < 0.001), depression (*p* < 0.001), and post-traumatic distress (*p* < 0.001) than males. Being employed in a COVID-19 unit, being transferred to other units, and living with children and the elderly were factors associated with higher levels of psychological distress. Greater coping abilities were detected in physicians, and in those HCWs employed in COVID units. Our findings evidenced that the psychological consequences of the pandemic were non-homogeneously distributed across HCWs categories and pointed out the presence of specific in-hospital and out-of-hospital risk factors.

## 1. Introduction

Coronavirus disease 2019 (COVID-19) is an ongoing pandemic condition caused by Severe Acute Respiratory Syndrome Coronavirus-2 (SARS-CoV-2), first detected in China in 2019 and rapidly spread worldwide. Since February 2020, Italy has been strongly hit by the COVID-19 pandemic, with a higher fatality rate than other countries [1], which required the adoption of extraordinary measures to limit viral transmission [2].

Alongside adopting restrictive rules to limit social interactions and movements, the COVID-19 outbreak required a profound and rapid reorganization of the hospital system, with an overall redefinition of clinical units, spaces, and work teams [3,4]. Healthcare workers (HCWs) have been faced with several stressful circumstances related to the pandemic, linked to both in-hospital factors—such as moving to COVID-19 dedicated clinical units—and the increased risk for being infected and potentially transmitting the SARS-CoV-2 to familiars, patients, and co-workers [5]. According to previous research on other infectious diseases such as SARS and Ebola outbreaks, HCWs are likely to experience a high psychological burden from epidemic situations [6,7]. This can be due to multiple factors, including the increased workload, the risk of nosocomial transmission, and the need to make difficult decisions on the rationing of care delivery. Consistently with these observations, the studies that already evaluated the psychological consequences of the COVID-19 pandemic on HCWs highlighted increased levels of anxiety, depression, insomnia, and distress [6,8,9]. Interestingly, they also revealed that sex differences and professional roles could explain different psychological responses to the pandemic situation. Females displayed a higher prevalence rate of depression and anxiety, while nurses were more exposed to the same symptoms when compared to medical doctors [6]. Moreover, the composition of the family unit, with the presence of elderly and/or children, could represent a factor that increases the fear of being infected or infecting by COVID-19 and, more in general, the risk for anxiety symptoms and psychological distress. These observations suggest that the possibility of identifying the demographic, hospital, or extra-hospital risk factors that can contribute to a greater psychological vulnerability in HCWs during the COVID-19 pandemic is of considerable importance to effectively design psychological help services and to be able to properly plan prevention and intervention strategies. Interestingly, observations from previous epidemics such as SARS-CoV-1 and Ebola evidenced that coping resources played an important role in protecting HCWs from psychological distress. Still, data on differences in resilience abilities among HCWs categories during the COVID-19 outbreak are currently lacking [10].

Therefore, the present study aimed to evaluate the psychological outcome among HCWs during the COVID-19 pandemic, addressing general psychopathological outcomes, resilient abilities, and COVID-19 specifically-related symptoms. Furthermore, the role of in-hospital (i.e., professional role, clinical unit) and extra-hospital (i.e., the composition of the familiar nucleus) risk factors were examined.

## 2. Materials and Methods

### 2.1. Study Design and Participants

This study is a cross-sectional, hospital-based online anonymous survey conducted during June 2020 among the HCWs of the University Hospital of Padua. The peak of total confirmed cases in the province of Padova occurred between the end of March and the first weeks of April (250 affected patients admitted in non-ICU and 55 in ICU wards in the Hospital of Padova) [11]. A recruitment email was sent to all the directors and head-nurses and they forwarded it to all healthcare professionals who provided direct patient care: 1154 physicians, 2505 nurses and other sanitary professionals, and 1219 healthcare assistants. The email explained the reason for the study. Participants self-administered the survey using an anonymous Google Suite^®^ survey link made available for a period of 4 weeks.

### 2.2. Demographic and Clinical Data

In the survey, participants had to report several demographic data (Table 1), including the composition of their family nucleus, their occupational category (physicians, nurse or other sanitary professionals, healthcare assistants), their place of work during the emergency (working in a COVID-19 dedicated unit or being transferred to such a Unit), and personal or indirect contact with COVID-19 infection (family members, colleagues).

The survey contained questions assessing, on a scale from 0 to 10, the perception of personal risk (“How much do you feel in danger for COVID-19?”), the concerns about the possibility of infecting others (“To what extent do you feel concerned about infecting any of your family members?”), and the level of attention paid to physical symptoms (“In the last period, do you pay more attention to physical symptoms than usual?”). Four questions also investigated, on a scale from 0 to 10, the satisfaction with the professional choice, the level of motivation to go to work, and the perceived quality of the work environment and of the relationships with colleagues, before and after the COVID-19 outbreak. Some self-reported questionnaires were included in the survey to assess psychological distress, specific fears, and resilience coping: (1) the Fear of COVID-19 questionnaire [12] to assess specific fears and anxiety about the COVID-19 pandemic, (2) the 7-item Generalized Anxiety Disorder (GAD-7) scale [13] to assess generalized anxiety, (3) the 9-item Patient Health Questionnaire (PHQ-9) [14] to measure depressive symptoms, (4) the 7-item Insomnia Severity Index (ISI) [15] to assess insomnia, (5) the 22-item Impact of Event Scale-Revised (IES-R) [16] to assess post-traumatic symptoms, and (6) the Brief Resilient Coping Scale (BRCS) [17] to measure the tendencies to cope with stress in a highly adaptive way. As in the study by Lai et al. (2020), the cutoff scores for detecting symptoms of major depression (PHQ-9), anxiety (GAD-7), insomnia (ISI), and post-traumatic distress (IES-R) were 10, 7, 15, and 26, respectively [18,19,20,21]. Participants who had scores greater than the cutoff threshold were defined as having severe symptoms. The internal consistency of all the scales was satisfactory, ranging from 0.87 and 0.96.

### 2.3. Statistical Analysis

All statistical analyses were conducted with the software IBM SPSS Statistics (version 25, Armonk, NY, USA). To investigate differences between groups a series of ANOVAs were performed. To investigate the effects of the COVID-19 pandemic on work environment satisfaction, repeated measures ANOVAs were performed using the four questions investigating satisfaction with the professional choice, motivation to go to work, perceived quality of work environment, and relationships with colleagues, as the repeated measures factors and the professional role as the between-subjects factor. Multiple regression analyses were performed to assess the influence of putative demographic predictors on psychopathological symptoms and resilience coping abilities. All the analyses were controlled for age and sex when appropriate.

## 3. Results

Nine hundred and ninety-six HCWs of the Hospital of Padua completed the survey and were included in the study. The main characteristics of the sample are reported in Table 1. The response rate of physicians was 22% for males and 21% for females. Nurses and other sanitary professionals participated with a response rate of 15% in males and 19% in females, whereas for healthcare assistants the response rate was 5% in males and 15% in females.

### 3.1. Psychological Distress

Among the whole sample, 597 participants reported anxiety symptoms (59.9%; severe symptoms: *n* = 97; 9.7%, cut-off score: 7), 421 reported depressive symptoms (42.3%; severe symptoms: *n* = 37; 3.7%, cut-off score: 10), 424 reported symptoms of insomnia (42.6%; severe insomnia: *n* = 34; 3.4%, cut-off score: 15), and 652 reported symptoms of post-traumatic distress (65.5%; severe symptoms: *n* = 97; 9.7%, cut-off score: 26). Table 2 and Table 3 report psychopathological scores based on occupational roles and sex. As compared to physicians, nurses and other sanitary professionals displayed higher perception of personal risk, higher scores at the fear of COVID-19 questionnaire, and higher levels of post-traumatic distress symptoms. Compared to healthcare assistants, nurses and other sanitary professionals reported significantly higher levels of depression. Moreover, compared to both physicians and healthcare assistants, nurses and other non-medical health professionals showed higher levels of insomnia and generalized anxiety. Being female, was associated with increased concerns about the possibility of infecting others with COVID-19 and greater levels of generalized anxiety, COVID-19 related fears, depression, and post-traumatic distress symptoms (Table 3).

Table 4 reports psychopathological scores based on being employed in a COVID-19 dedicated unit and on being transferred to another unit. Being employed in a COVID-19 dedicated unit was associated with an increased perception of risk about COVID infection, higher fear of infecting others, higher attention paid to physical symptoms, and higher depression levels and post-traumatic distress symptoms. No significant associations were observed for anxiety symptoms, insomnia symptoms, and specific fears as measured by the Fear of Covid questionnaire when compared to those who were not employed in a COVID-19 dedicated unit. Being transferred to another unit during the emergency period was associated with higher anxiety levels, higher depression scores, higher insomnia, and higher post-traumatic distress symptoms when compared to those who were not transferred to another unit. No significant associations were observed for the perception of risk about COVID-19 infection, concerns about infecting others, attention paid to physical symptoms, and scores on the Fear of Covid questionnaire. Being infected or having a family member or a colleague who has been infected by COVID-19 was not associated with any difference in questionnaires’ scores, with the exception of higher depression levels in healthcare workers infected by COVID-19 (PHQ: 7.4 ± 5.0 vs. 4.9 ± 4.6; F (3992) = 5.21; *p* = 0.023) and higher post-traumatic distress symptoms (IES: 19.7 ± 16.6 vs. 16.8 ± 15.7; F (3992) = 10.19; *p* = 0.001) and depression (PHQ: 5.1 ± 4.5 vs. 4.7 ± 4.7; F (3992) = 4.38; *p* = 0.037) associated with COVID infection in a colleague. Living with school-aged children was associated with higher perception of risk about COVID-19 infection, higher concerns about infecting others, higher scores on the Fear of Covid Questionnaire, higher levels of anxiety, and higher levels of post-traumatic distress symptoms when compared to those who were not living with school-aged children. Living with elderly family members was associated with higher perception of risk about COVID-19 infection, higher concerns about infecting others, higher scores at the Fear of COVID questionnaire, and higher anxiety levels when compared to those who were not living with elderly. Table 5 reports psychopathological scores based on living with school-aged children and on living with elderly. Living with children aged less than 5 was not associated with differences in any psychopathological scale when compared to those who were not living with children aged less than 5 years.

We performed a series of multiple regression models to better identify the predictors of psychopathological symptoms, including age, sex, professional role, working in a COVID Unit, being transferred to a different unit during the emergency, and living with school-age children or elderly family members as putative predictors. Significant predictors for higher scores on the fear of COVID questionnaire were being female, not being a physician, living with elderly family members, and living with school-aged children; predictors of higher anxiety symptoms (GAD-7) were: being female, being a nurse, being transferred to a different unit during COVID-19 emergency, living with elderly family member and living with school-aged children; predictors of higher depressive symptoms (PHQ-9) were: being female, being a nurse and being transferred to a different unit during the emergency; predictors of higher levels of insomnia (ISI) were: being a nurse and being transferred to a different unit during the emergency; predictors of greater post traumatic distress symptoms (IES-R) levels were: being female, being transferred to a different unit during the emergency, and living with school-aged children. Results of regression models are reported in Table 6.

### 3.2. Changes in Work Environment Satisfaction Levels

The repeated measures ANOVAs showed a significant main effect of time on the quality of relationships with the colleagues (F (1993) = 4.62, *p* = 0.032), with health care workers reporting a significant worsening in their relationships with colleagues following the COVID-19 outbreak. In addition, a significant main effect of professional role on all the examined variables was evidenced: satisfaction with the professional choice (F (2, 993) = 5.06, *p* = 0.006), motivation to go to work (F (2993) = 15.33, *p* < 0.001), perceived quality of the work environment (F (2993) = 18.33, *p* < 0.001), and perceived quality of relationships with colleagues (F (2993) = 3.00, *p* = 0.050). The analyses revealed that nurses and other sanitary professionals, compared to healthcare assistants, reported generally lower levels of satisfaction with the professional choice and quality of relationships with the colleagues, and, compared to both healthcare assistant and physicians, lower motivation to go to work. Healthcare assistants, instead, reported a general greater satisfaction with the work environment compared to both physicians and nurses and other sanitary professionals. A significant interaction between professional role and time was present for the perceived quality of the work environment (F (2993) = 4.20, *p* = 0.015). Compared to physicians and healthcare assistants, nurses and other sanitary professionals displayed a greater reduction in satisfaction with the work environment following the COVID-19 outbreak.

### 3.3. Resilience Coping Abilities

In our sample of health care professionals, we observed greater resilient coping abilities in physicians, as compared to nurses and other sanitary professionals, and in males in comparison with females (Table 2 and Table 3). Health care workers employed in a COVID Unit displayed significantly greater coping abilities while being transferred to a different unit during the emergency was not associated with any difference in the brief resilient scale (Table 4). Coping abilities in health care workers were mildly negatively correlated with scores at the Fear of COVID questionnaire (rho = −0.12; *p* < 0.001), anxiety (GAD: rho = −0.15; *p* < 0.001), depression (PHQ: rho = −0.14; *p* < 0.001), and insomnia (rho = −0.15; *p* < 0.001), but not with post-traumatic distress symptoms (rho = −0.029; *p* = 0.364). However, in a multiple regression model, being a physician (beta = 0.072; t = 2.06; *p* = 0.040) and working in a COVID unit (beta = 0.116; t = 3.59; *p* < 0.001) were identified as the main predictors of coping abilities with no additional contribution provided by psychological symptoms.

## 4. Discussion

The present research adds to previous studies addressing the psychological well-being of HCWs who faced the COVID-19 related emergency by investigating general psychopathology (i.e., anxiety, depression, post-traumatic distress) and COVID-19 specific symptoms in a large sample of subjects working in an Italian tertiary Hospital (*n* = 996). Within the whole sample, most of the participants were female (75.8%) and were employed as non-medical health professionals (63.8%). In line with previous observations, female subjects reported higher psychopathological symptoms than males, with increased anxiety, depression, and distress scores [22]. These differences can be explained by the already established sex differences in depression and anxiety prevalence rates [23], but also by the increased susceptibility women have shown to the negative psychological consequences of the COVID-19 pandemic [24,25]. In line with these observations, we also evidenced that females reported higher COVID-19 related fears and concern about the possibility of infecting others, which is probably related to their role as family caregivers [26,27].

As compared to physicians, nurses and other sanitary professionals displayed more severe anxiety, insomnia, post-traumatic distress symptoms, fear of COVID-19, and a higher perception of personal risk. Additionally, compared to healthcare assistants, they reported higher levels of depression. It is interesting to note that these results confirmed previous studies conducted on non-European samples, thus supporting the presence of transcultural risk factors related to the pandemic that mainly impact the nursing staff [22,28]. Among them, it has been speculated that the greater worsening in psychopathological scores reported by nurses compared to other HCW categories may be related to their more intense contacts and exposure to patients suffering [29]. Nevertheless, factors related to the work organization are also likely to be involved [30]. Our results highlighted that following the COVID 19 outbreak, nurses showed a greater reduction in workplace satisfaction than physicians and healthcare assistants thus supporting the presence of factors inherent to the work organization that may strike this category more than the others, and which deserve to be further investigated. In addition to factors belonging to the hospital environment, our study highlighted that out-of-hospital factors, such as being the caregiver of children and elderly, may play a role in the emergence of psychological difficulties. Indeed, being a caregiver was associated with increased levels of anxiety, post-traumatic distress symptoms, fear of COVID-19, and higher concerns about infecting others. These findings are in line with previous observations highlighting the impact of familiar difficulties on the psychological wellbeing of caregivers during the pandemic [31], but should be specifically evaluated on healthcare workers. Indeed, during the first pandemic wave in Italy (the period during which this study was conducted), healthcare workers were subjected to both a high workload and an increased caregiving burden due to both the health consequences of the pandemic and the lockdown of most of the productive activities and schools. In line with this, our findings indicated that living with elderly family members and with school-aged children predicted fear of COVID-19, generalized anxiety, and post-traumatic distress scores, thus confirming an increased psychological risk in those HCWs who have a caregiving role.

An aspect that was proposed to be relevant in determining the psychological consequences of the pandemic is the ability to effectively cope with its consequences [32,33]. Interestingly, our results evidenced that healthcare workers employed in a COVID-19 dedicated unit displayed greater coping abilities than others. We can speculate that this may be due to an effective and powerful sense of teamwork that was established during the first wave of the pandemic, which helped in actively facing the multiple adversities and stressors. Moreover, since social support has been demonstrated to bolster coping abilities and to positively influence the resilience of healthcare workers during the pandemic, we can hypothesize that the sense of gratitude conveyed by both media and society during the first phases of the COVID-19 outbreak may have been helpful in determining a sense of effectiveness in the healthcare teams [34]. However, given the notable changes that pandemic control measures have over time [35], coupled with the ambiguity inherent in some media-conveyed messages [36], further and longitudinal evaluations of the influence of social support on coping abilities of HCWs are needed. The observation that physicians have shown superior coping skills compared to nurses and other health professionals confirms that HCWs should not be considered a homogeneous population in the psychological impact they have as a result of the pandemic. Overall, these observations indicate the need to further study the role of specific coping strategies in facing the pandemic consequences among HCWs to design targeted and more effective treatment strategies [37]. A recently published meta-analysis [7] stated that HCWs usually reported mild depression and anxiety symptoms, with a prevalence rate of full depression and anxiety diagnosis similar to the one that the general population displayed during the same time. The absence of a control group does not allow us any inference on these observations; nevertheless, it is worth noting that a non-negligible proportion of our participants reported severe psychiatric symptoms, with percentage rates similar to the ones reported by Lai and colleagues (2020). Given these observations, together with the fact that HCWs are likely to be particularly susceptible to COVID-19 related distress, our results are likely to highlight the importance of early detecting sub-threshold symptoms, as well as the need of activating mental-help services to prevent the risk of evolution in severe, complex, and enduring psychopathology.

This study should be considered in light of several limitations. First, the cross-sectional nature of the observations does not allow to distinguish preexisting psychological symptoms and newly onset ones. Furthermore, the absence of a longitudinal follow-up does not allow to know the evolution of psychological symptoms. Second, the fact that all the participants were recruited in one hospital is likely to limit the generalization of the results. Third, the absence of a control group does not allow any conclusive inference about the risk and severity of psychological symptoms in HCWs compared to the general population. Fourth, the data regarding work environment satisfaction were assessed retrospectively and must be taken with caution, since the current satisfaction could be a bias towards recollection of previous satisfaction. Fifth, the present research evaluated the prevalence of some specific psychological symptoms using self-administered questionnaires. Therefore, these data are not corroborated by structured diagnostic interviews, and no deductions can be drawn about the presence of specific psychiatric diagnoses.

## 5. Conclusions

In conclusion, our findings highlighted differences in the specific psychopathological severity by different HCWs categories and sex, with nurses and females being most vulnerable to the psychological consequences of the COVID-19 pandemic. Furthermore, our results confirmed that having caregiving roles is likely to influence the psychological impact of the pandemic, and suggest the need of further investigating the role of coping resources in mediating the psychological impact of the pandemic among HCWs. Overall, the present research highlighted the presence of multiple risk and vulnerability factors to the pandemic’s psychological consequences, which can help in recognizing those HCWs that have a higher risk for psychological distress when planning specific healthcare re-organization programs and dedicated psychological interventions.

## Figures and Tables

**Table 1:** Demographics.

Demographic Variables	N	%
Overall	996	100
Sex		
Men	241	24.2
Women	755	75.8
Age		
20–29	90	9.0
30–39	197	19.8
40–49	290	29.1
>50	419	42.1
Family composition		
Alone	127	12.8
Children under 12 years	232	23.3
Elderly over 70 years	89	8.9
Children + elderly	19	1.9
Other	529	53.1
Professional Role		
Physicians	215	21.6
Nurses and other health professionals	635	63.8
Healthcare assistants	146	14.7
Transferred to a different unit (yes)	141	14.2
Working in a COVID-19 unit (yes)	420	42.2
Positive for COVID-19 infection (yes)	17	1.7
A family member positive for COVID-19 infection (yes)	19	1.9
A colleague positive for COVID-19 infection (yes)	500	50.2

**Table 2:** Differences in psychopathological scores by occupational role.

Psychopathological Variables	Physicians (P, *n* = 215) Mean (SD)	Healthcare Assistants (HA, *n* = 146) Mean (SD)	Nurses (N, *n* = 635) Mean (SD)	ANOVA (3992) F (*p*)	Post-Hoc Bonferroni
Perception of personal risk	5.13 (2.00)	5.37 (2.58)	5.65 (2.19)	**4.61 (0.010)**	N > P
Fear of infecting others	6.62 (2.45)	6.52 (3.03)	6.90 (2.59)	0.90 (0.408)	
Attention to physical symptoms	5.73 (2.62)	6.14 (2.86)	6.19 (2.67)	2.08 (0.126)	
Fear of COVID questionnaire	12.83 (4.58)	14.49 (5.55)	14.51 (5.66)	**5.15 (0.006)**	N > P
GAD-7	5.35 (4.50)	6.12 (4.54)	7.27 (5.50)	**7.64 (0.001)**	N > P, HA
PHQ-9	4.00 (3.94)	4.10 (4.04)	5.43 (4.81)	**6.35 (0.002)**	N > HA
ISI	6.49 (5.37)	6.62 (5.62)	8.29 (6.54)	**8.80 (<0.001)**	N > P, HA
IES-R	15.27 (14.93)	17.95 (15.26)	20.05 (16.92)	**4.23 (0.015)**	N > P
BRCS	13.71 (3.30)	12.84 (3.55)	12.85 (3.19)	**3.16 (0.043)**	N < P

GAD-7: 7-item Generalized Anxiety Disorder scale; PHQ-9: 9-item Patient Health Questionnaire; ISI: Insomnia Severity Index; IES-R: Impact of Event Scale-Revised; BRCS: Brief Resilient Coping Scale. Significant *p* values are indicated in bold (significance considered *p* < 0.05).

**Table 3:** Psychopathological scores in healthcare workers by sex.

Psychopathological Variables	Female (*n* = 755) Mean (SD)	Male (*n* = 241) Mean (SD)	ANOVA (3992) F (*p*)
Perception of personal risk	5.53 (2.20)	5.39 (2.30)	0.68 (0.410)
Fear of infecting others	6.88 (2.60)	6.47 (2.72)	**4.00 (0.046)**
Attention to physical symptoms	6.08 (2.72)	6.09 (2.63)	0.02 (0.880)
Fear of COVID scale	14.48 (5.53)	13.08 (5.14)	**12.68 (<0.001)**
GAD-7	7.03 (5.26)	5.63 (4.96)	**12.83 (<0.001)**
PHQ-9	5.26 (4.70)	3.90 (3.99)	**15.52 (<0.001)**
ISI	7.86 (6.29)	7.03 (5.99)	3.31 (0.069)
IES-R	19.80 (16.29)	15.29 (16.19)	**14.18 (<0.001)**
BRCS	12.88 (3.32)	13.51 (3.14)	**6.34 (0.012)**

GAD-7: 7-item Generalized Anxiety Disorder scale; PHQ-9: 9-item Patient Health Questionnaire; ISI: Insomnia Severity Index; IES-R: Impact of Event Scale-Revised; BRCS: Brief Resilient Coping Scale. Significant *p* values are indicated in bold (significance considered *p* < 0.05).

**Table 4:** Psychopathological scores in healthcare workers (HCWs) by unit and transfer to other units.

Psychopathological Variables	COVID Unit (*n* = 420) Mean (SD)	Not COVID Unit (*n* = 576) Mean (SD)	ANOVA (3992) F *(p)*	Transferred (*n* = 141) Means (SD)	Not Transferred (*n* = 855) Mean (SD)	ANOVA (3992) F (*p*)
Perception of personal risk	5.70 (2.28)	5.34 (2.17)	**6.52 (0.011)**	5.54 (2.47)	5.49 (2.18)	0.07 (0.796)
Fear of infecting others	7.01 (2.62)	6.62 (2.64)	**5.34 (0.021)**	7.08 (2.85)	6.73 (2.59)	1.91 (0.167)
Attention to physical symptoms	6.40 (2.63)	5.85 (2.72)	**9.77 (0.002)**	6.49 (2.64)	6.02 (2.70)	3.62 (0.057)
Fear of COVID questionnaire	14.19 (5.35)	14.11 (5.55)	0.21 (0.649)	14.60 (5.94)	14.07 (5.38)	1.14 (0.286)
GAD-7	6.91 (5.23)	6.52 (5.23)	1.61 (0.205)	8.04 (5.65)	6.47 (5.12)	**10.86 (0.001)**
PHQ-9	5.29 (4.51)	4.67 (4.61)	**4.91 (0.027)**	6.22 (5.06)	4.72 (4.46)	**12.98 (<0.001)**
ISI	7.86 (6.29)	7.51 (6.17)	0.98 (0.323)	8.94 (7.21)	7.44 (6.02)	**7.06 (0.008)**
IES-R	20.12 (16.65)	17.68 (16.11)	**6.40 (0.012)**	23.72 (17.69)	17.88 (16.01)	**15.65 (<0.001)**
BRCS	13.45 (3.15)	12.73 (3.35)	**11.43 (0.001)**	13.06 (3.14)	13.03 (3.31)	0.02 (0.903)

GAD-7: 7-item Generalized Anxiety Disorder scale; PHQ-9: 9-item Patient Health Questionnaire; ISI: Insomnia Severity Index; IES-R: Impact of Event Scale-Revised; BRCS: Brief Resilient Coping Scale. Significant *p* values are indicated in bold (significance considered *p* < 0.05).

**Table 5:** Psychopathological scores in HCWs by living with school-aged children and elderly.

Psychopathological Variables	Children (School-Aged) (*n* = 174) Mean (SD)	Not Children (*n* = 822) Mean (SD)	ANOVA (3992) F (*p*)	Elderly (*n* = 108) Means (SD)	Not Elderly (*n* = 888) Mean (SD)	ANOVA (3992) F (*p*)
Perception of personal risk	5.81 (2.19)	5.43 (2.23)	**4.57 (0.033)**	5.94 (2.58)	5.44 (2.17)	**4.87 (0.027)**
Fear of infecting others	7.24 (2.49)	6.69 (2.65)	**6.75 (0.010)**	7.42 (2.69)	6.71 (2.61)	**8.04 (0.005)**
Attention to physical symptoms	6.41 (2.64)	6.01 (2.70)	2.90 (0.089)	6.14 (2.99)	6.08 (2.66)	0.23 (0.630)
Fear of COVID questionnaire	14.78 (5.42)	14.01 (5.47)	**3.96 (0.047)**	15.67 (6.65)	13.96 (5.28)	**7.47 (0.006)**
GAD-7	7.48 (5.34)	6.52 (5.19)	**6.00 (0.014)**	7.70 (5.77)	6.57 (5.14)	**4.30 (0.038)**
PHQ-9	5.30 (4.86)	4.85 (4.51)	1.96 (0.161)	5.50 (4.78)	4.86 (4.55)	1.94 (0.164)
ISI	8.07 (6.36)	7.57 (6.20)	1.21 (0.271)	7.85 (6.45)	7.63 (6.20)	0.04 (0.852)
IES-R	21.01 (18.09)	18.22 (15.96)	**5.45 (0.020)**	21.63 (18.17)	18.35 (16.12)	2.89 (0.090)
BRCS	13.40 (3.25)	12.96 (3.29)	2.21 (0.138)	13.10 (3.36)	13.03 (3.28)	0.09 (0.769)

GAD-7: 7-item Generalized Anxiety Disorder scale; PHQ-9: 9-item Patient Health Questionnaire; ISI: Insomnia Severity Index; IES-R: Impact of Event Scale-Revised; BRCS: Brief Resilient Coping Scale. Significant *p* values are indicated in bold (significance considered *p* < 0.05).

**Table 6:** Predictors of psychopathological symptoms.

Independent Variables	Fear of COVID			GAD-7			PHQ-9			ISI			IES-R		
	B	t	*p*	B	t	*p*	B	t	*p*	B	t	*p*	B	t	*p*
Sex	0.07	2.07	**0.039**	0.08	2.42	**0.016**	0.11	3.34	**0.001**	0.03	0.93	0.355	0.1	2.87	**0.004**
Age	0.03	1.74	0.082	−0.01	−0.15	0.88	−0.04	−1.14	0.256	0.05	1.46	0.144	0.04	1.19	0.239
Living with children	0.07	2.35	**0.019**	0.09	2.82	**0.005**	0.05	1.64	0.101	0.04	1.37	0.171	0.08	2.61	**0.009**
Living with elderly	0.09	2.7	**0.007**	0.06	2.03	**0.042**	0.04	1.32	0.186	0	0.07	0.946	0.05	1.66	0.098
Being transferred	0.02	0.65	0.519	0.09	2.73	**0.006**	0.09	2.91	**0.004**	0.07	2.16	**0.031**	0.1	3.15	**0.002**
Working in a COVID unit	0.01	0.21	0.834	0.02	0.49	0.622	0.05	1.39	0.164	0.01	0.41	0.682	0.05	1.61	0.108
Being a nurse	0.02	0.49	0.624	0.11	2.55	**0.011**	0.14	3.17	**0.002**	0.14	3.18	**0.002**	0.08	1.76	0.079
Being a phyisicians	−0.09	−2.05	**0.041**	−0.02	−0.44	0.659	0.05	1.03	0.303	0.01	0.14	0.888	−0.02	−0.45	0.657
Model	R^2^	F	*p*	R^2^	F	*p*	R^2^	F	*p*	R^2^	F	*p*	R^2^	F	*p*
	0.04	4.71	**<0.001**	0.05	6.4	**<0.001**	0.05	6.47	**<0.001**	0.03	3.6	**<0.001**	0.05	6.15	**<0.001**

GAD-7: 7-item Generalized Anxiety Disorder scale; PHQ-9: 9-item Patient Health Questionnaire; ISI: Insomnia Severity Index; IES-R: Impact of Event Scale-Revised. Significant *p* values are indicated in bold (significance considered *p* < 0.05).

## Data Availability

Data available on request from the corresponding author.
